# Flexible Nanofiber Pressure Sensors with Hydrophobic Properties for Wearable Electronics

**DOI:** 10.3390/ma17102463

**Published:** 2024-05-20

**Authors:** Yang Liu, Baoxiu Wang, Jiapeng Chen, Min Zhu, Zhenlin Jiang

**Affiliations:** 1 College of Chemistry and Chemical Engineering, Research Center for Advanced Mirco- and Nano-Fabrication Materials, Shanghai University of Engineering Science, Shanghai 201620, China; m340121127@sues.edu.cn (Y.L.); 34210007@sues.edu.cn (J.C.); zhu0304min@163.com (M.Z.); 2 Key Laboratory of High Performance Fibers & Products, Ministry of Education, Donghua University, Shanghai 201620, China; 3 College of Chemical Engineering, Nanjing Tech University, Nanjing 210009, China

**Keywords:** pressure sensor, human healthcare monitoring, bacterial cellulose nanofiber, nanofiber aerogel

## Abstract

In recent years, flexible pressure sensors have received considerable attention for their potential applications in health monitoring and human–machine interfaces. However, the development of flexible pressure sensors with excellent sensitivity performance and a variety of advantageous characteristics remains a significant challenge. In this paper, a high-performance flexible piezoresistive pressure sensor, BC/ZnO, is developed with a sensitive element consisting of bacterial cellulose (BC) nanofibrous aerogel modified by ZnO nanorods. The BC/ZnO pressure sensor exhibits excellent mechanical and hydrophobic properties, as well as a high sensitivity of −15.93 kPa^−1^ and a wide range of detection pressure (0.3–20 kPa), fast response (300 ms), and good cyclic durability (>1000). Furthermore, the sensor exhibits excellent sensing performance in real-time monitoring of a wide range of human behaviors, including mass movements and subtle physiological signals.

## 1. Introduction

Flexible pressure sensors have attracted widespread attention in various promising application areas such as human health monitoring [1], artificial skin [2], intelligent robotics [3], and touchable electronic devices [4]. According to the sensing mechanism, the reported pressure sensors are usually categorized into piezoresistive [5], piezoelectric [6], capacitive [7], and electrical friction sensors [8]. Among these pressure sensors, piezoresistive pressure sensors are widely used because of their simple structure [9], high-pressure sensitivity [10], fast response [11], easy signal processing [9], reliability [12], and durability [13]. In recent times, there have been numerous attempts to design flexible piezoresistive pressure sensors that are more sensitive, have a faster response time, and have a wide operating range [14]. Based on different process designs and material innovations, a growing number of manufacturing techniques are now available for the production of polymer strain/pressure sensors [15,16]. However, when the sensors are used for human body detection, it is difficult to satisfy the high sensitivity, fast response, and wide operating range with waterproof performance.

Flexible piezoresistive pressure sensors typically employ a variety of elastic polymers as flexible substrates [17], including polydimethylsiloxane [18], polyurethane [19], polyvinyl alcohol [20], and other similar materials. Additionally, conductive nanomaterials such as graphene [21], carbon nanotubes [22], silver nanowires [23], and others are incorporated to enhance the conductivity of the substrate. Among the materials used in sensor preparation, zinc oxide (ZnO) plays a significant role due to its piezoelectric properties [24]. Currently, ZnO produced by the hydrothermal reaction method [25], the carbo-thermal transport growth method [26], and the electron beam evaporation [27] have a complete morphology and structure. In addition to the selection of suitable substrates and conductive fillers, the structural design of the materials is also important for the preparation of high-performance pressure sensors [28,29]. Yan [30] rationally designed and implemented a piezoresistive pressure sensor based on an ultra-sensitive bionic MXene by molding the microstructure of Ginkgo biloba. The obtained pressure sensor exhibits an ultra-high sensitivity of 403.46 kPa^−1^, a short response time of 99.3 ms, and a remarkable durability of 12,000 load-unload cycles. Xia [31] presents a piezoresistive tactile sensor strategy based on indium tin oxide (ITO) nanocrystals and plant fiber composites. The pressure sensor exhibits a wide detection range (0–100 kPa), high sensitivity (464.88 kPa^−1^), fast response time (6.93 ms) and recovery time (7.18 ms), and good loading and unloading stability. Qin [32] proposed a flexible pressure sensor with a “sandwich” structure, which employs patterned wood fiber as the substrate and MXene as the sensitive layer. The sensor exhibits high sensitivity (1417.9 kPa^−1^) and a fast response time (30.77 ms) at pressures below 100 kPa. However, these sensors lack hydrophobicity, and exposure to water can lead to short circuits that can interfere with use.

**Figure 1:**
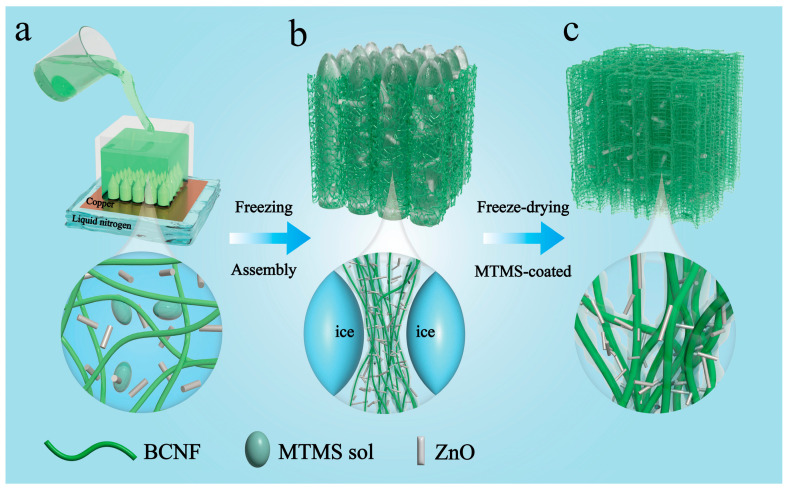
Design and construction principle of BC/ZnO nanocomposite fiber aerogel: (**a**–**c**) Schematic diagram of nano-composite fiber aerogel preparation.

In this paper, a simple freeze-drying method for the preparation of bacterial cellulose/zinc oxide (BC/ZnO) aerogels is presented, and a schematic diagram of the fabrication process is shown in Figure 1. BC was chosen as the backbone of the aerogel due to the advantages of biocompatibility, non-toxicity, low cost, and easy plasticity. Methyltrimethoxysilane (MTMS) was used as the cross-linking agent, and the MTMS structure contained one hydrophobic Si-CH and three hydrolyzable Si-OCH, which could endow the nanofibers with certain hydrophobic properties. Meanwhile, in order to improve the conductive contact points of the porous aerogel, rod-shaped zinc oxide was introduced into the BC skeleton to form a cross-linked structure. The resulting BC/ZnO aerogels were superhydrophobic with a water contact angle (WCA) of 130° and exhibited excellent compressibility and resilience. The aerogel piezoresistive pressure sensor exhibited high sensitivity, fast response, and good repeatability. The sensor was successfully used as a wearable electronic device to monitor various human movements. The conductive and superhydrophobic aerogels prepared in this work are simple and inexpensive, and even in water or humid environments, aerogels have great potential for a wide range of applications, such as wearable devices, e-skins, and artificial intelligence.

## 2. Experimental

### 2.1. Materials

Bacterial cellulose nanofibers (BCNF) suspension was obtained from Guilin Qihong Technology Co., Ltd. (Guilin, China). Sodium hydroxide (NaOH), anhydrous ethanol, zinc acetate dihydrate (Zn(CH_3_COO)_2_·2H_2_O), methyltrimethoxysilane (MTMS), and acetic acid were purchased from Sinopharm Chemical Reagent Co., Ltd. (Shanghai, China).

### 2.2. Preparation of BC/ZnO Aerogel

Rod-shaped ZnO was prepared by hydrothermal synthesis [33]. Initially, 0.438 g of Zn(CH_3_COO)_2_·2H_2_O was dissolved in 20 mL of anhydrous ethanol, and 1.6 g of NaOH was dissolved in 40 mL of anhydrous ethanol. After stirring at room temperature for two hours, the two solutions were mixed and further stirred for two hours. The resulting solution was then transferred to a 100 mL PTFE-lined autoclave. Subsequently, the PTFE was sealed and placed in an oven for hydrothermal treatment at 150 degrees Celsius for 24 h. After the reaction, the PTFE was allowed to cool naturally. The solution was filtered and washed several times in deionized water and alcohol. The ZnO powder was obtained after drying in an oven at 60 °C for 12 h. The dispersion was then dried in an oven at 60 °C. The reaction equations are as follows:

(1) $$\mathrm{NaOH}\to {\mathrm{Na}}^{+}+{\mathrm{OH}}^{-}$$

(2) $$\mathrm{Zn}{\left({\mathrm{CH}}_{3}\mathrm{COO}\right)}_{2}\to {\mathrm{Zn}}^{2+}+2{\mathrm{CH}}_{3}{\mathrm{COO}}^{-}$$

(3) $${\mathrm{Zn}}^{2+}+2{\mathrm{OH}}^{-}\to \mathrm{ZnO}+H_{2}O$$

Initially, the dispersion was diluted to a mass fraction of 0.3 wt% BC dispersion. Subsequently, different mass fractions (0.3 wt%, 0.4 wt%, 0.5 wt%, 0.6 wt%) of ZnO powder were added to the dispersion. A solution of 5 × 10^−3^ mol L^−1^ acetic acid (6 mL) was prepared in water (2 mL) and 2 mL of MTMS was added. This was allowed to react for 30 min to obtain a completely hydrolyzed MTMS silica sol. We took 500 μL of MTMS sol and added it to 8 g of BC dispersion of a certain concentration. This was then stirred for 1 h and sonicated for 0.5 h. The well-dispersed solution was poured into the pre-prepared molds and placed on a copper plate. The sample was then subjected to directional freezing using liquid nitrogen at a temperature of −196 °C. Following this, the sample was placed in a vacuum freeze dryer for a period of 48 h. Finally, the freeze-dried aerogels were stored in an air-filled environment for three days to facilitate the complete bonding of the MTMS within the aerogel.

### 2.3. Preparation of BC/ZnO Aerogel-Based Pressure Sensors

To assemble the aerogel pressure sensor, the aerogel was cut into strips (20 mm long, 20 mm wide, and 5 mm thick). Two pieces of aluminum foil were fixed as two electrodes at both ends of the aerogel, the copper foil was used as a wire to extend it out, and the outer layer was wrapped by a polyimide film as an isolation layer to obtain a stable signal output. Polyimide (PI) film has good insulating properties and is suitable for insulating materials for various electrical appliances [34]. The real-time output signal of the sensor was recorded by Keithley 2400.

### 2.4. Characterization

The sample morphology was observed by scanning electron microscopy (SEM, SU8000, Hitachi, Tokyo, Japan). The SEM was operated at a voltage of 5 kV and a working distance of 8.5 mm and coated with a thin gold layer before SEM observation. The sample was tested by X-ray diffraction (XRD, Rigaku Ultimate IV, RIKEN, Tokyo, Japan) at angles from 5° to 90°, operating at a scan rate of 5 °/min under a Cu target at 40 kV and 40 mA current. The chemical structure of the samples was analyzed by Fourier transform infrared spectroscopy (FT-IR, AVATAR370, PerkinElmer Instruments Ltd., Waltham, MA, USA) in the range of 4000–500 cm^−1^ with a resolution of 4 cm^−1^. The mechanical properties of the aerogel were tested using a universal testing machine (AGX-V, Shimadzu, Tokyo, Japan). Contact angle tests were measured at room temperature using an optical contact angle meter (OCA 20 Micro, DataPhysics Instruments, Beijing, China). We used an electrochemical workstation to test LSV curves (CHI600E, CH Instruments, Shanghai, China) and measured the performance of pressure sensors using a high precision digital source meter (Keithley 2400, Portland, OR, USA). The fiber structure of the aerogel was observed by atomic force microscopy with a scanning range of 5 μm (AFM, Bruker Dimension ICON).

## 3. Results and Discussion

### 3.1. Microstructure and Mechanical Properties of Aerogels

Figure 1a–c illustrates the fabrication routes and strategies employed for the production of BC/ZnO aerogels. MTMS contains a hydrophobic group (Si-OH) [35]. The dehydration and mineralization of Si-OH can be employed to facilitate the formation of a polymethylsilsesquioxane (PMSQ) coating on the surface of BC fibers [36]. During freezing, the nanofibers and MTMS are subjected to a pushing force from moving ice crystals, which leads to the formation of pore structures (Figure 1b) [37]. After freeze-drying, the ice crystals are removed, and the formed PMSQ is encapsulated on the surface of the nanofibers. PMSQ serves to impart hydrophobic properties to the fiber, while simultaneously acting as a cross-linking agent for the fiber [38]. Therefore, BC/ZnO aerogels are obtained directly by freeze-drying.

**Figure 2:**
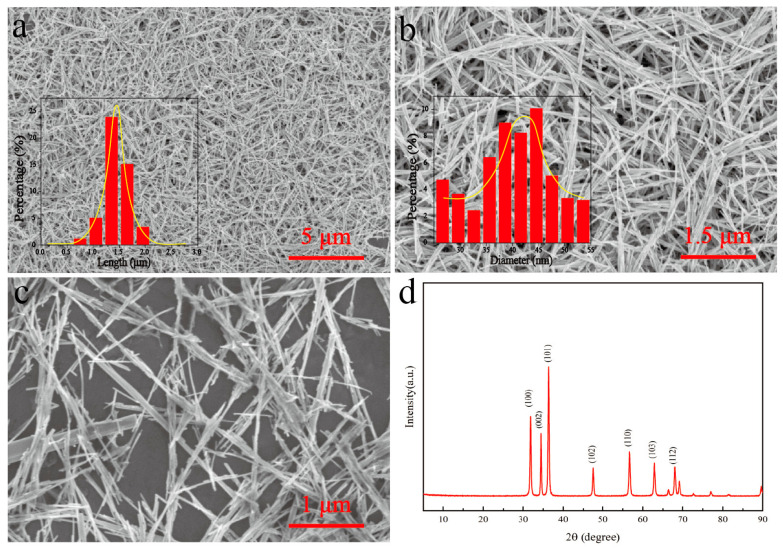
(**a**–**c**) SEM images of ZnO nanorods; insets show the length and diameter particle size distribution of ZnO nanorods. (**d**) XRD images of ZnO nanorods.

The morphology of the ZnO nanorods was observed by SEM, as illustrated in Figure 2a–c. The length and diameter particle size distributions of ZnO were subjected to statistical analysis using Nano Measurer, with the results presented in the inset. The synthesized ZnO nanorods exhibited a uniform morphology with a large aspect ratio, a length of approximately 1.5 μm, and a diameter of approximately 49 nm. Figure 2d depicts the XRD diffractograms of the ZnO nanorods, with diffraction angles 2θ of 31.7°, 34.4°, 36.3°, 47.5°, 56.6°, 62.9°, 67.9°, and 69.1°; all the diffraction peaks correspond to the ZnO structure (PDF card no. 89-0510).

**Figure 3:**
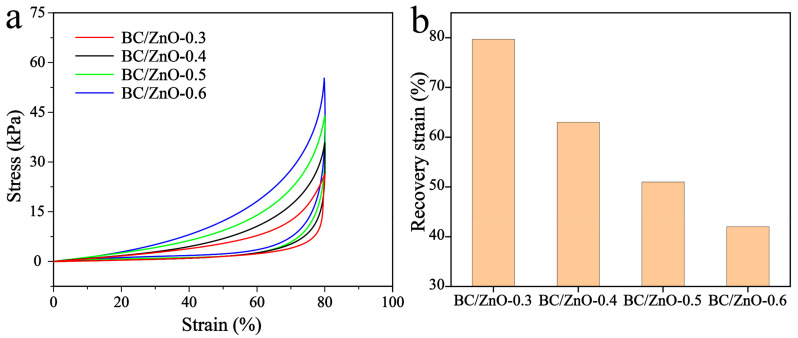
BC/ZnO-0.3, BC/ZnO-0.4, BC/ZnO-0.5, and BC/ZnO-0.6 aerogels: (**a**) compression stress–strain ratio curves and (**b**) recovery strain after 80% compression of aerogel.

The BC/ZnO aerogels obtained with different contents (0.3 wt%, 0.4 wt%, 0.5 wt%, and 0.6 wt%) of ZnO were designated as BC/ZnO-0.3, BC/ZnO-0.4, BC/ZnO-0.5, and BC/ZnO-0.6. As the concentration of ZnO nanorods in BC/ZnO-0.4, BC/ZnO-0.5, and BC/ZnO-0.6 gradually increases, the compression recovery performance of these materials gradually decreases. The concentration of ZnO nanorods may be too high for the MTMS silica sol to support the recovery of the aerogel after it is subjected to pressure, resulting in a weaker aerogel recovery, as illustrated in Figure 3. Among the samples, BC/ZnO-0.3, with a concentration of 0.3 wt% ZnO nanorods, exhibited the most favorable recovery performance after compression. Therefore, this concentration was selected as a reference for subsequent experiments.

**Figure 4:**
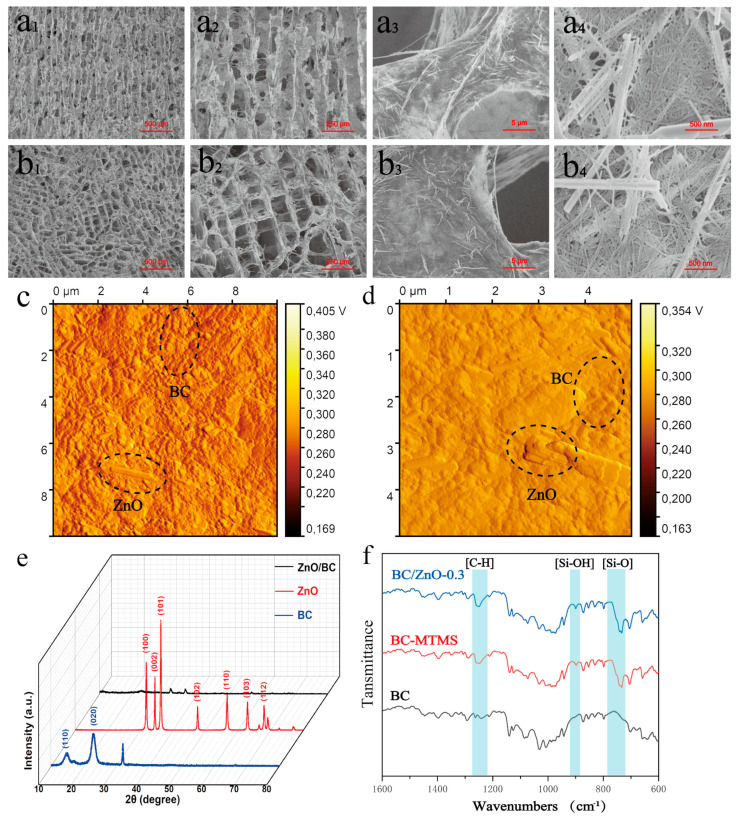
Nanocomposite BC/ZnO-0.3 aerogel microstructures: (**a_1_**–**a_4_**) longitudinal SEM pore structures at different magnifications and (**b_1_**–**b_4_**) transverse SEM pore structures at different magnifications. (**c**,**d**) AFM image of BC/ZnO-0.3. (**e**) XRD spectra of BC, ZnO and BC/ZnO-0.3 aerogel. (**f**) FTIR spectra of BC, BC-MTMS and nanocomposite BC/ZnO-0.3 aerogels.

The longitudinal structure of the BC/ZnO-0.3 aerogel is shown in Figure 4a,b. The BC/ZnO-0.3 aerogel has a cytosolic network structure and an obviously oriented pore structure. The pore aperture is approximately 100 μm, parallel to the direction of ice growth. The pore wall of the BC/ZnO-0.3 aerogel is formed by BC nanofibers tightly entangled with ZnO nanorods and a large number of ZnO nanorods. The uniform intertwining of the nanorods provides the basis for the formation of the subsequent pressure sensors. Figure 4b_1_–b_3_ illustrates the transverse structure of the BC/ZnO-0.3 aerogel, which reveals a regular pore structure with homogeneous pores in the shape of a square. We characterized BC, ZnO, and BC/ZnO-0.3 aerogels by XRD (Figure 4c). The diffraction images of BC at 2θ = 14.3°, 22.7° corresponding to the (110), (020) crystal planes show type I cellulose structure. The BC/ZnO-0.3 aerogel exhibits peaks of BC and ZnO. The FTIR of aerogel is shown in Figure 4d. The main characteristic peaks of BC were concentrated at 3343 cm^−1^, 2897 cm^−1^, 1432 cm^−1^, and 1153 cm^−1^ and closely related to the stretching vibration of -OH, the stretching vibration of -CH_2_, the bending vibration of C-H, and C-O-C in the β-glycosidic bond, respectively. In comparison to BC, the BC/ZnO-0.3 exhibited vibrational contraction peaks at 1272 cm^−1^, which corresponded to C-H, and the characteristic peaks at 950–860 cm^−1^, which corresponded to Si-OH. These peaks were indicative of the structural units Si-CH_3_ and Si-OH, respectively [39]. These structures were formed by the hydrolysis and polycondensation of MTMS. The presence of Si-OH is indicative of the double-silica-bridged polymer network structure, suggesting that the hydrolysis and mineralization of MTMS formed an incomplete condensation network wrapped around the BC fibers.

**Figure 5:**
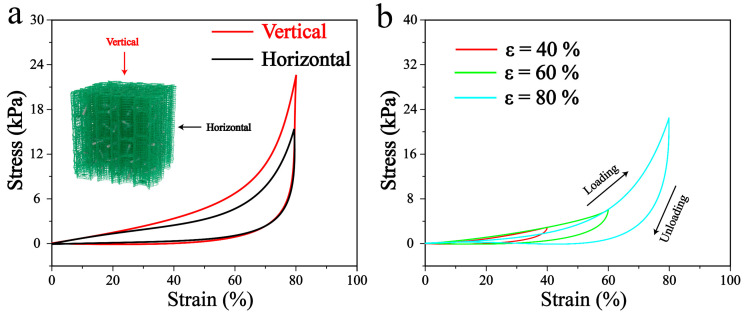
(**a**) Comparison of transverse and longitudinal mechanical properties of BC/ZnO-0.3 aerogel. (**b**) Mechanical properties of BC/ZnO-0.3 aerogel in compression at 40%, 60%, and 80%.

The BC/ZnO-0.3 aerogel exhibits excellent mechanical strength and compression resilience properties due to its multi-scale structure, comprising both micron-scale and nanoscale components. As illustrated in Figure 5a, the pore structure aligned parallel to the ice crystal growth direction is designated as the vertical direction, while the pore structure perpendicular to the ice crystal growth direction is designated as the horizontal direction. Upon application of 80% compressive strain in the longitudinal and transverse directions, respectively, the compressive strain of the nanocomposite fiber aerogel demonstrated recoverability. However, due to the pore orientation, the transverse compression exhibited a higher recovery performance compared to the longitudinal compression. Figure 5b illustrates the compressive stress–strain behavior of BC/ZnO-0.3 aerogel. The compressive strain is linear elastic deformation when the compressive strain is less than 10%. When the compressive strain is between 10% and 60%, the material pore collapses to form a stress plateau. The stress gradually increases as the compressive strain increases. When the compressive strain is greater than 60%, the internal pore structure of the material is densified, and the mechanical strength is rapidly increased. The strain recovery of BC/ZnO-0.3 is such that after the strain recovery, the aerogel can still keep its original shape without rupture or deformation.

### 3.2. Sensing Performance and Mechanism

**Figure 6:**
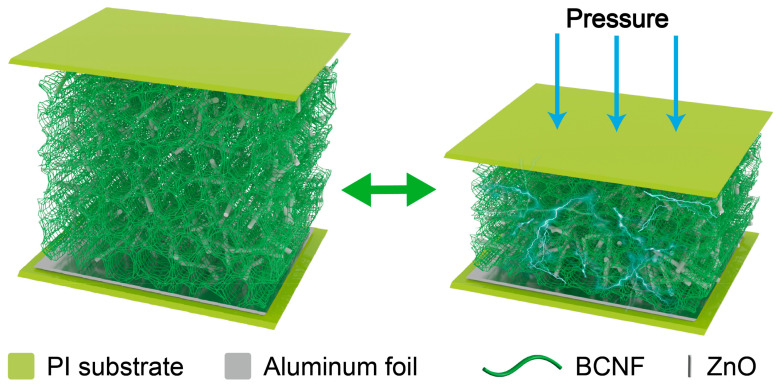
BC/ZnO aerogel sensing schematic diagram.

The schematic diagram for the pressure sensor is shown in Figure 6. When subjected to pressure, the aerogel deforms, allowing the ZnO to come into contact with each other to form new sensing pathways. The increase in the number of neighboring sensing pathways leads to a decrease in the overall resistance of the aerogel. When the external force is withdrawn, the BC aerogel springs back, returning the resistance of the aerogel to its initial state.

**Figure 7:**
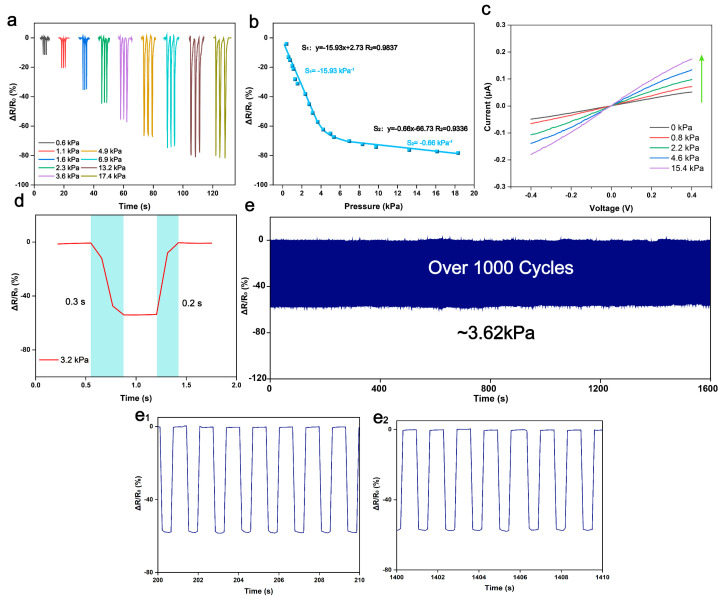
BC/ZnO aerogel sensing performance. (**a**) The relative resistance change in BC/ZnO-0.3 at different pressures. (**b**) The relative resistivity change versus different pressures is presented in fitted curves for BC/ZnO-0.3. (**c**) Current–voltage curves of BC/ZnO-0.3 at different pressures. (**d**) Response and recovery time of the sensor at 3.2 kPa pressure. (**e**) Relative resistance change in pressure sensor at 3.62 kPa pressure for 1000 cycles of loading/unloading. (**e_1_**,**e_2_**) Amplified response curves for 200 s–210 s and 1400 s–1410 s cycle tests.

The sensing characteristics of the BC/ZnO-0.3 sensor were evaluated using a stepper motor in conjunction with a pressure tester, as illustrated in Figure 7a. Upon increasing the applied pressure from 0.6 kPa to 17.4 kPa, the resistance change rate was observed to be 80.3%, with the resistance gradually reaching a saturation point beyond 17.4 kPa pressure. Pressure sensitivity (S) is defined as (R−R_0_)/(R_0_P), where R_0_ and R represent the resistance values in the initial state and at a certain applied pressure, respectively, and P is the pressure. The resistance change rate obtained by the sensor at different pressures was measured and averaged over several measurements. The data were then curve fitted at 0.25–4 kPa and 4–20 kPa to obtain the sensitivities of S_1_ and S_2_, respectively. As shown in Figure 7b, the BC/ZnO exhibits a high sensitivity of −15.93 kPa^−1^ over a wide range of 0.25–4 kPa for a small pressure range. In the high-pressure range of 4–20 kPa, the pressure sensitivity is −0.66 kPa^−1^, a consequence of the significant reduction in the accessible points of ZnO under high pressure. Figure 7c illustrates the current–voltage (I–V) curves at different pressures (0–15.4 kPa), demonstrating high linearity and optimal ohmic contact between the BC/ZnO electrodes. As is well known, response time represents a crucial metric for assessing the sensing performance of pressure sensors [40]. Response time is usually defined as the difference between the time of the start of pressure application and the final steady state time. As shown in Figure 7d, a rapid response time under 3.2 kPa pressure was obtained to be 300 ms and a recovery time of 200 ms. In addition, repeated loading/unloading cycles at a pressure of 3.62 kPa did not result in waveform changes, indicating excellent stability and durability (Figure 7e).

### 3.3. Applications for Pressure Sensors

**Figure 8:**
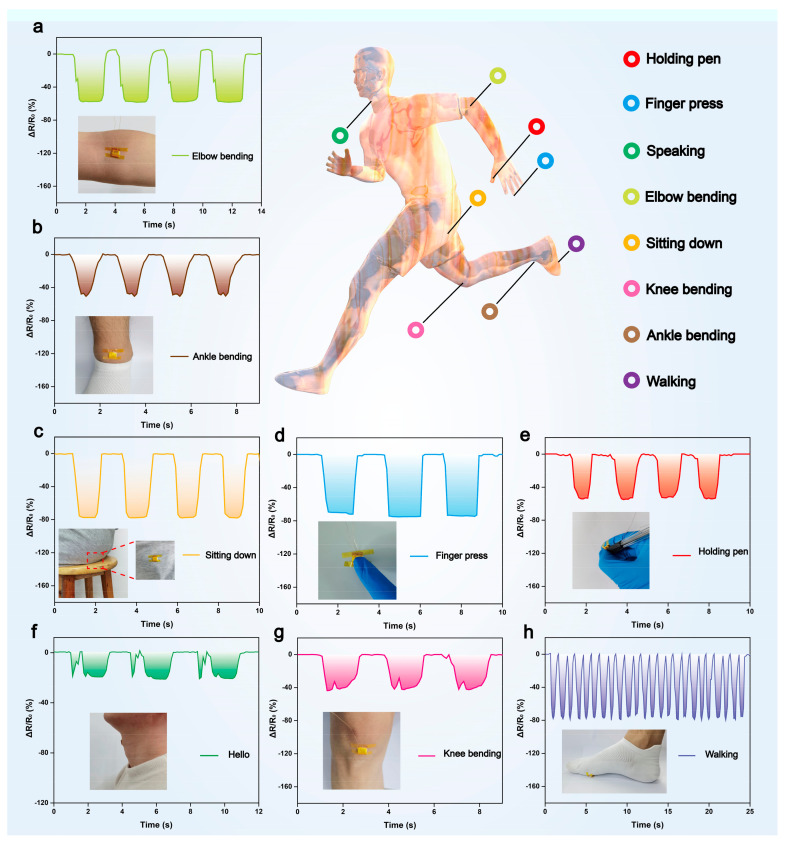
(**a**–**h**) Response curves for tester’s elbow bending, ankle bending, sitting down, finger pressing, holding pen, speaking, knee bending, and walking maneuvers in a motion monitoring test.

The proposed pressure sensor is distinguished by its high sensitivity, extensive measurement range, reliable response, and consistent repeatability, rendering it suitable for a multitude of applications. Our tests have demonstrated that the pressure sensor can be utilized to detect a diverse array of human activity signals, including sound monitoring, knee and wrist bending, and strenuous movements. First, as illustrated in Figure 8a–d, the response curves of the BC/ZnO pressure sensor to the tester’s elbow, ankle, sitting and standing, and finger presses are presented. Additionally, real-time monitoring of behaviors during motion, including pen grip, pronation, knee bend, and walking (Figure 8e–h), was conducted. It can be observed that the response waveforms for each movement are distinctive, stable, repetitive, and easily distinguishable. The integration of BC/ZnO pressure sensors with machine learning algorithms and other technologies has the potential to enable the realization of functions such as gait recognition and motion monitoring. These sensors have significant application prospects in the fields of healthcare, wearable electronic devices, and intelligent robot development, among others.

### 3.4. Hydrophobic Properties of Pressure Sensors

**Figure 9:**
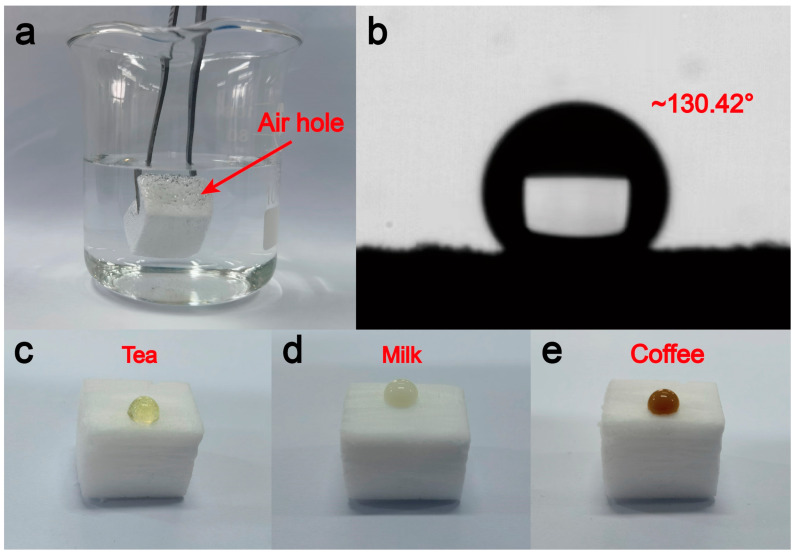
Hydrophobic properties of aerogel pressure sensors: (**a**) image of submerged water, (**b**) indicating hydrophobic angle, (**c**) tea stain, (**d**) milk, and (**e**) coffee.

Pressure sensors with excellent hydrophobic properties are capable of effectively preventing the intrusion and adsorption of liquids and can function stably in a liquid environment and accurately sense pressure changes. A pressure sensor with good hydrophobicity can extend the service life of the pressure sensor and improve its reliability and stability. When the aerogel is immersed in water, air bubbles densely surround the aerogel, demonstrating good hydrophobicity (Figure 9a). The contact angle of the aerogel is 130.42° (Figure 9b). The aerogel demonstrated not only hydrophobicity to water but also to a range of liquids, including milk, tea, coffee, and others. The liquids in question exhibit good fluidity on the surface of the aerogel, which effectively resists corrosion and adsorption of liquids on the sensor, thereby ensuring the long-term stability and reliability of the sensor (Figure 9c–e).

## 4. Conclusions

In conclusion, 3-D porous BC/ZnO aerogel pressure sensors were prepared by directional freezing and freeze-drying. The ice crystal extrusion formation during the preparation process altered the aerogel structure, while the cross-linking agent imparted the aerogel with excellent waterproof performance. The excellent spatial structure of the aerogel enables the prepared pressure sensor to exhibit excellent sensitivity and pressure detection range. In the pressure range of 0.25–4 kPa, the sensor exhibits a sensitivity of −15.93 kPa^−1^, while in the high-pressure range of 4–20 kPa, the sensitivity is −0.66 kPa^−1^. The pressure sensor exhibits a fast response time (300 ms) and a fast recovery time (200 ms) and a good cycling performance (>1000). Furthermore, the sensor’s exceptional hydrophobicity (contact angle of 130.42°) minimizes fluid contamination and erosion during operation. With their low cost, ease of use, and simple processing, BC/ZnO aerogel-based pressure sensors are expected to play an important role in future human–computer interaction, electronic skin, and smart wearable devices.

## Data Availability

Data are contained within the article.
